# Harnessing the Power of Quality Assurance Data: Can We Use Statistical Modeling for Quality Risk Assessment of Clinical Trials?

**DOI:** 10.1007/s43441-020-00147-x

**Published:** 2020-03-30

**Authors:** Björn Koneswarakantha, Timothé Ménard, Donato Rolo, Yves Barmaz, Rich Bowling

**Affiliations:** 1 F. Hoffmann-La Roche, Basel, Switzerland; 2 Roche Products Ltd, Welwyn Garden City, UK; 3 Genentech Inc., A member of the Roche Group, South San Francisco, USA

**Keywords:** Quality assurance, Clinical trial, Advanced analytics, Statistical modeling, Good clinical practice (GCP), Audit

## Abstract

**Background:**

The increasing number of clinical trials and their complexity make it challenging to detect and identify clinical quality issues timely. Despite extensive sponsor audit programs and monitoring activities, issues related to data integrity, safety, sponsor oversight and patient consent have recurring audit and inspection findings. Recent developments in data management and IT systems allow statistical modeling to provide insights to clinical Quality Assurance (QA) professionals to help mitigate some of the key clinical quality issues more holistically and efficiently.

**Methods:**

We used findings from a curated data set from Roche/Genentech operational and quality assurance study data, covering a span of 8 years (2011–2018) and grouped them into 5 clinical impact factor categories, for which we modeled the risk with a logistic regression using hand crafted features.

**Results:**

We were able to train 5 interpretable, cross-validated models with several distinguished risk factors, many of which confirmed field observations of our quality professionals. Our models were able to reliably predict a decrease in risk by 12–44%, with 2–8 coefficients each, despite a low signal-to-noise ratio in our data set.

**Conclusion:**

We proposed a modeling strategy that could provide insights to clinical QA professionals to help them mitigate key clinical quality issues (e.g., safety, consent, data integrity) in a more sustained data-driven way, thus turning the traditional reactive approach to a more proactive monitoring and alerting approach. Also, we are calling for cross-sponsors collaborations and data sharing to improve and further validate the use of statistical models in clinical QA.

**Electronic supplementary material:**

The online version of this article (10.1007/s43441-020-00147-x) contains supplementary material, which is available to authorized users.

## Background

Compliance with the fundamental principles of good clinical practice (GCP) ensures the rights, safety and well-being of research subjects and ensures the integrity of clinical research data. Trial sponsors are required by the International Conference on Harmonization (ICH) guidelines to implement and maintain Quality assurance (QA) and quality control systems to achieve these objectives [1].

Traditional clinical QA practices heavily rely on audits to detect sites or studies with quality issues [2]. Audit programs usually follow a risk-based approach hence all studies cannot be covered. Furthermore, audits often report on issues that have already occurred. The increasing number of clinical trials and sites and the growing complexity of study designs make it challenging to detect and identify clinical quality issues timely. Traditional site monitoring strategies, which rely on on-site visits with source data verification (SDV) and on risk-based approaches, are also attempting to mitigate the risk of occurrence of clinical quality issues [3, 4]. However, despite extensive audit programs and monitoring activities performed by sponsors, issues related to data integrity, safety, sponsor oversight, protection of primary endpoints and patient consent are recurring audit and inspection findings [5, 6].

Innovation (e.g., use of "artificial intelligence") in study set-up and design has been focused on operational and scientific aspects of clinical trials such as patient recruitment or optimal study design [7]. Quality by design principles for clinical trials are applied by several sponsors [8], yet they follow a standard approach (i.e., check-lists) and do not leverage on statistical modeling or similar techniques.

A holistic and data-driven approach for QA that could help anticipating and reducing risk of occurrence of key clinical quality issues (data integrity, safety, sponsor oversight, protection of primary endpoints and patient consent) and that could also be used for clinical trial quality by design is not currently available. However, the industry has recently been trying to leverage modern developments in data management and IT systems that facilitate the cross-analysis of clinical studies. Statistical analysis can be performed on these data based on certain attributes to help identify issues and to be able to estimate the quality risk, where we define risk as the probability of an issue to occur and its severity. We used our combined, historical clinical studies and quality assurance data from Roche/Genentech to explore the feasibility of a statistical model. We proposed a modeling strategy that could provide insights to clinical QA professionals to help them mitigate some of the key clinical quality issues (e.g., safety, consent, data integrity) more holistically and efficiently, thus turning the traditional reactive approach to more of a proactive monitoring and alerting approach. We also highlighted the constraints of using advanced analytics in the context of clinical study risk assessment, as a number of quality issues remain unpredictable, due to the potential randomness and noise inherent to clinical and operational study data (see “Interpretation” and “Limitations” sections).

The development of a statistical model that can help anticipating clinical trial quality issues requires a deep understanding of data science, clinical trials and QA. The project has been conducted by the Roche/Genentech Quality Analytics and Insights team, a team of data analysts and data scientists, in collaboration with Roche/Genentech clinical and QA subject matter experts (SMEs).

The mission of the Roche/Genentech quality analytics team is to build data-driven solutions for clinical QA at Roche/Genentech to complement and augment traditional QA approaches to improve the quality and oversight of GCP and Good Pharmacovigilance Practices (GVP) regulated activities.

## Methods

### Prerequisites

To estimate quality risk during clinical trials, we relied on the outcomes of investigator site audits and inspections. There was a potential bias in this approach as only a fraction of investigator sites participating in a clinical trial were being audited or inspected during the course of a clinical study. Thus, any risk that we could estimate excludes risks that had not been regularly detected in the past.

Quality risk modeling for clinical study has a solid business use case, hence it was essential that the resulting model was interpretable and that the identified risk factors were usable for our stakeholders, i.e., Roche Quality Program Leads (QPLs) and Molecule Strategy team.

The clinical quality assurance data (i.e., individual quality issues) were reported as audit/inspections findings and were labeled with categories, sub-categories and finding statements. The source database was the Roche/Genentech audit finding management tool. To translate the quality issues into areas that could be interpreted across sponsors (while directly linking to key GCP requirements), we mapped all the individual findings statement to defined Clinical Impact Factors (CIF). See Table 1 for the consolidated list of CIF considered in our analysis.

**Table 1.** List of Clinical Impact Factors.

Area	Clinical impact factor
Human subject protection	Informed consent
Human subject protection	Safety
Reliability of trials results	Data integrity
Reliability of trials results	Protecting primary endpoints
Reliability of trials results	Sponsor oversight

### Data

The data used in this project came from Roche/Genentech Product Development clinical quality and study operational data, collected over 8 years. Our data set consisted of 4100 individual findings, which had been reported from investigator site audits and inspections between the years 2011–2018. On average, 86.7 audits and/or inspections were conducted each year, the inspections making up ~ 13% of all quality activities. A typical audit or inspection generates around 5.9 findings on average. To identify quality risk factors for clinical studies, we complemented our data set with study attributes and operational study data. For a list of all initial features, see the Electronic Supplementary Material (ESM).

### Modeling Approach

A single audit or inspection can result in one or more findings of a given impact factor of different classification. However, we determined with our stakeholders that it is either the absence or the presence of any number of findings that is relevant for any CIF. We therefore decided to model the risk as the probability of having one or more findings associated with a given CIF, eventually breaking down the modeling problem to 5 individual binary classifications. The unit of analysis being a single audit or inspection activity and the modeling goal was to get a well-calibrated risk prediction and to isolate underlying risk factors rather than training an optimal classifier.

During the explorative modeling phase, classical applied statistical learning methods with a built-in feature selection process such as decision trees, random forest and lasso did not give satisfying results. Even though, modeling performance as measured by area under the receiver operator characteristics (ROC) curve (AUC) resulted in values between 0.5 and 0.65—which is low but distinguishable—these algorithms also generated models with comparable AUC values from a set of simulated features. Those had similar distributions as our original features, but could only have a coincidental correlation with our modeling target. This indicated a very low signal-to-noise ratio in our data set and that we could not rely on automated feature selection methods (as they are prone to integrate noise into the model, which does not allow us to adequately identify risk factors). We therefore decided to carefully engineer binary features that reflect sensible signals that we could detect using exploratory data analysis (EDA). We would then use these features to train logistic regression models using an iterative approach for selecting the best combination of features.

### Feature Engineering and Selection

We took a hypothesis-driven approach and generated binary features based on the outcome of EDA and the recommendations of quality subject matter experts. To account for non-linear relationships between our target outcome and continuous features we scaled, centered and normalized them using Yeo-Johnson power transformation [9] before binning them into 5 segments which would each be explored independently during EDA. To avoid including near zero variance predictors [10] single values of discrete features that applied to less than 5% of all audits and inspections were grouped. Features that showed an effect on the frequency of CIF findings and were not contradicting stakeholders expectations were binarized and considered for modeling. During the binarization process all missing values were replaced with ‘no.’

To control overfitting [11] we performed the final feature selection on a reduced training set with data from 2011 to 2015. We fitted an optimal logistic regression model for each clinical impact factor to the training data set iteratively, assessing different feature combinations aiming to reduce the Bayesian information criterion BIC [12]. We made sure that none of the included features were correlating with one another (strongest correlation observed was − 0.21) and checked for interactions. We identified one interaction between the features “randomization” and “study design,” which were then combined to the binary features “non-randomized parallel or sequential study” and “non-randomized single group study”. Further we calculated a site burden score that is reflecting how many other trials are run by Roche at a given site compared to all other sites within the same study. To this end we ranked all sites within a study by how many other Roche trials were ongoing at the same time in the same therapeutic area and converted that rank to a cumulative distribution rank to obtain values between 0 and 1. The higher the score value the more studies were running in parallel.

For the complete list of features, see ESM Table 1.

Last but not least we trained the final model on the entire data set using the previously identified features giving us the final coefficient values (see Fig. 2a).

### Modeling Validation and Calibration

**Figure 1.**
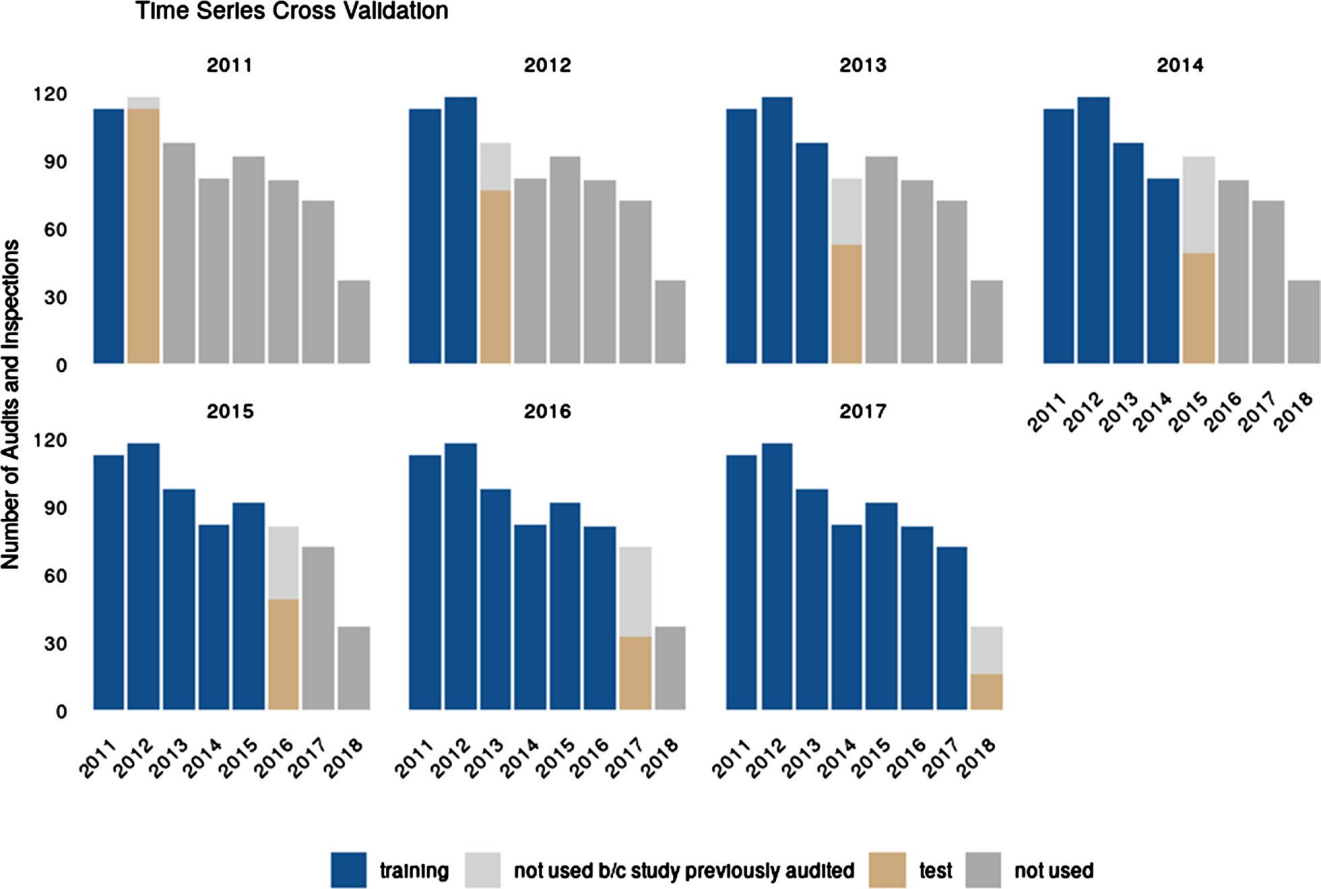
Visualization of Time Series Cross-Validation Strategy—To Validate Model Performance We Retrained Logistic Regression Models with a Previously Determined Features on Past Data (Blue) and Evaluated Performance on Next Years Data (beige) While Excluding Audits and Inspections of Studies that had Previously Been Audited (Light Gray). This process was repeated for each year from 2011 to 2017.

Historically, audits had been planned on an annual basis and risk assessment for site audit selection varied from year to year. Hence, we tested how well the quality risk of a given year can be predicted using all of the past data using a time series cross-validation strategy (see Fig. 1). In order not to overfit on study features, we excluded all audits and inspections of studies that had already been audited in the past [13]. For each of the resulting test sets we calculated AUC as a measure of discrimination and Brier Score as a measure of calibration (see Table 2).

**Table 2.** Mean Modeling Performance per CIF Model—Mean AUC and Brier Score Including Standard Error (SE) were Calculated Based on Test Set Predictions Derived from Time Series Cross-Validation Strategy with One Value per Year from 2011 to 2017.

CIF	Mean AUC ± SE	Mean Brier Score ± SE	Calibrated prediction range	Base rate probability (%)
Consent	0.63 ± 0.13	0.247 ± 0.004	37–55% (∆18%)	55
Data integrity	0.67 ± 0.14	0.174 ± 0.019	68–84% (∆12%)	84
Protecting primary endpoints	0.57 ± 0.07	0.199 ± 0.009	64–86% (∆22%)	86
Safety	0.65 ± 0.09	0.232 ± 0.008	15–59% (∆44%)	59
Sponsor oversight	0.55 ± 0.07	0.235 ± 0.009	40–66% (∆26%)	76

To obtain a well-calibrated model, it is advisable to recalibrate the model output using a calibration curve fitting observed vs predicted probabilities [14]. In our case the calibration of model outputs was important to define the upper and lower boundaries of the predicted probabilities. It is the underlying assumption of regression models that the risk represented by the coefficients is additive. However, for most audits and inspections in our data set only 1 (sometimes 2) coefficients applied, making it impossible to test whether the assumption of the additive nature of our models adequately represents our data.

For the calibration curve, the range of the predicted probabilities of the test sets of the cross-validation splits were equally divided into 4 bins. For each bin the observed probability and the 75% confidence intervals (CI75) were calculated using the frequency of the actually observed findings. To assess the quality of the calibration, we compared the CI75 to the base rate probability for each CIF finding. We deliberately set the calibration curve to the base rate probability if the CI75 and base rate were overlapping or merged the observations with a neighboring bin.

## Results

### Performance

**Fig. 2:**
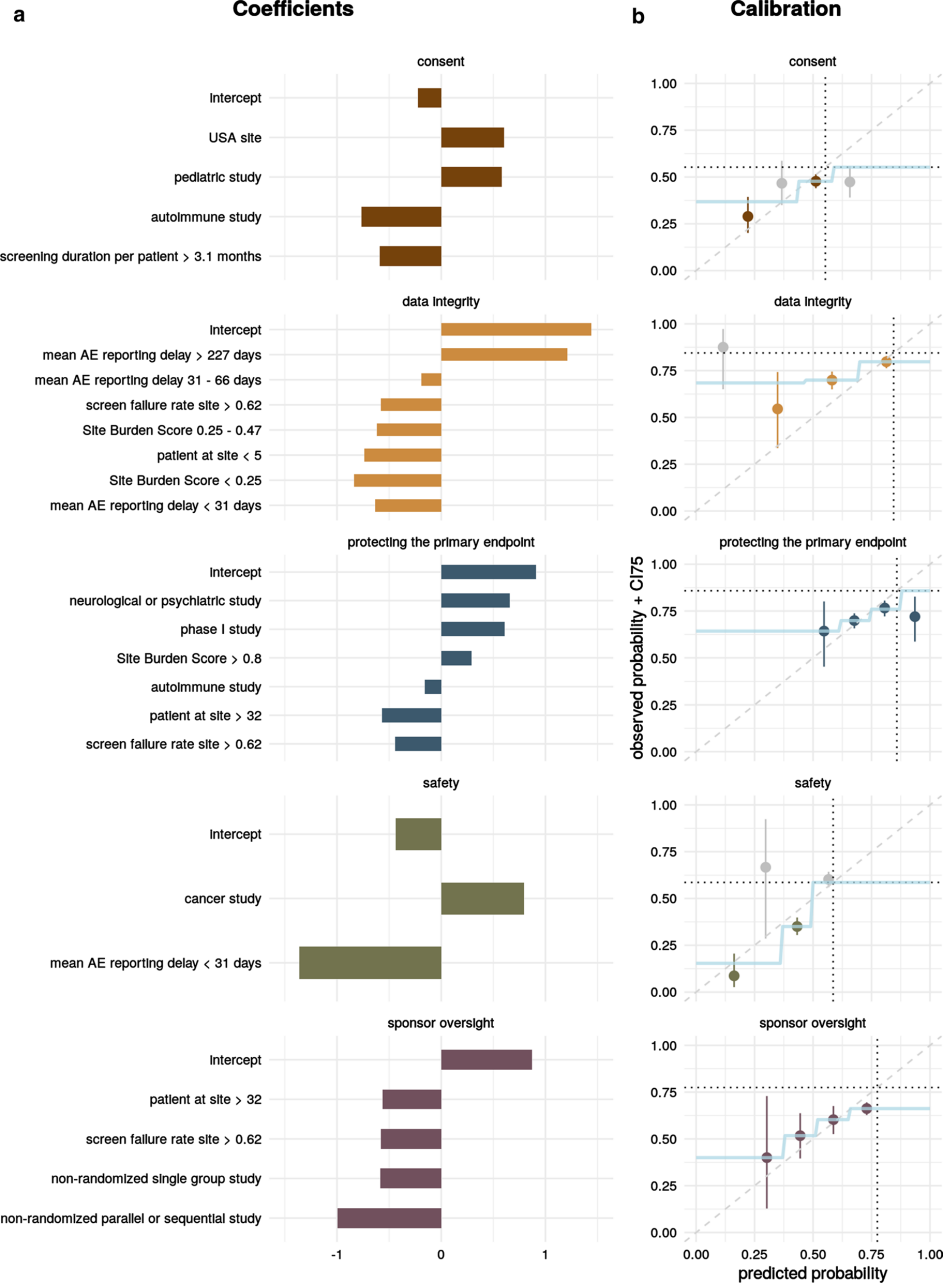
Model Performance. Coefficient Values were Obtained After Fitting Logistic Regression Models with a Previously Determined Set of Coefficients onto the Entire Data Set (**a**). After Dividing the Range of Predicted Probabilities for the Test Sets of the Time Series Cross-Validation Strategy into 4 Equal Segments the Observed Probabilities Including 75% Confidence Intervals (CI75) were Calculated Using the Frequency of Audits and Inspection with One or More Findings of the Indicated CIF (Each CIF is Indicated by One Color) Within a Segment. The Dotted Line Represents the Base Rate Probability. We then Modeled the Relationship Between the Predicted and Observed Values with a Step Function. If the CI75 of a Segment was Large (Overlapping with Base Rate), the Observations were Either Merged with a Neighboring Segment or the Fit Defaulted to Base Rate (**b**).

We were able to identify a set of features that would adequately model the probability of having at least one finding for all CIF (see Fig. 2a). We found that none of the models were able to sufficiently predict an increase of risk compared to base rate but all of them can make good estimates for the decrease of risk of 12–44% depending on the model (see Fig. 2b and Table 2) with 2–8 coefficients each (see Fig. 2a).

As performance metrics, mean AUC and Brier Score with standard error (SE) were measured on the test sets derived from the time series cross-validation strategy (see Table 2). Mean AUC values were between 0.55 and 0.67 which is low but distinguishable. Calibration quality as measured by the mean Brier Score (0 equals best and 1 equals worst calibration [15]) were between 0.174 and 0.247 showing adequate calibration. To check for biases introduced by our EDA-based feature engineering approach on the complete data set, we plotted individual coefficient and AUC values for each individual annual split (see ESM Fig. 1a, b) not detecting concernable differences for values generated from the holdout period (2016–2018) and the feature selection period (2011–2015). We determined coefficient values were relatively stable from 2014 onward meaning that the signal represented by the features was time period independent (see ESM Fig. 1a).

The calibration aims to primarily provide upper and lower boundaries for the model output. The delta (∆) indicates the difference between those upper and lower limits. For comparison the base rate probability for having one or more findings of a specific impact factor in the entire data set is also given.

### Interpretation

#### Clinical Impact Factor: Consent

The consent CIF model suggests an increased risk for sites within the US and pediatric studies and a decreased risk for autoimmune studies and sites with a long active screening period compared to the number of patients enrolled. For pediatric studies, the informed consent process required the signature of both parents which provided a reasonable explanation for the model behavior. Unfortunately, we could not offer a process-related explanation for all of the coefficients. However, we could confirm that they did not contradict the everyday observations by our Quality Program Leads (QPLs).

#### Clinical Impact Factor: Data Integrity

The risk factors of the data integrity CIF model on the other hand seemed to be very intuitive. Sites with less than 5 patients, with a high screen failure rate, that run fewer studies in parallel and whose AEs were reported timely after the AE onset date had a lower risk of having a finding in that category. None of these features were process-related but they were sensible intuitive proxies for the data processing burden at the site.

#### Clinical Impact Factor: Safety

The safety CIF model was very simple with only two features: whether the study in question was a cancer study and if the AEs were reported in a timely manner. Typically, patients in cancer studies had a lot more AEs than patients from other studies and most GCP findings that fell into that category were related to the AE reporting process. Meaning that both features were very process-centric. For further investigation on clinical trial safety reporting, other approaches using machine learning for anomaly detection [16, 17] had demonstrated value and might be more suitable.

#### Clinical Impact Factor: Sponsor Oversight

The risk for sponsor oversight findings was reduced for non-randomized studies; among those it was decreased the most for studies with parallel or sequential study design, compared to those with single group design. Additionally, the risk decreased for sites with a high screen failure rate (> 0.62) and if the site had enrolled more than 32 patients. Here we found a mix of process-related features such as randomization and study design and proxy features such as site enrollment.

#### Clinical Impact Factor: Protecting the Primary Endpoints

We identified phase I, neurological and psychiatric studies to be at greater risk; while the risk decreased for autoimmune studies. Sites that conducted multiple Roche studies in parallel were at greater risk. Sites with a high screen failure rate or that were high enrollers had a lower risk. For this model, all final features seemed to be non-process-related proxies.

### Deployment

The models outputs were displayed in the form of a cockpit/dashboard made available to Roche/Genentech QPLs. The outputs are used to complement ongoing risk assessment methods, as we acknowledge the current limitations of the model (see section below).

## Discussion

Despite some obvious limitations and challenges which we will discuss further below, we are able to propose a model for each of the CIFs. Using time series cross-validation, we verified that the signal our models were detecting was independent of time periods and thus did not seem to be influenced by changes of the business auditing strategy (see ESM Fig. 1a, b). We could further identify some of the features as being directly related to the underlying processes but we needed to acknowledge that most of them were merely proxy measurements not revealing the root cause of the quality issue. Last but not least, we found that automated feature selection processes such as lasso did not work in a low signal-to-noise ratio and that we obtained better results by handpicking the logistic regression features to obtain interpretable reasonably well-calibrated models. Altogether, this work builds a good foundation for future risk modeling endeavors as more process-related data can be collected and analyzed.

### Limitations

Even though having gathered data over many years, we found the signal indicating quality findings within our data set to be very weak. Increasing the size of the training data set during time series cross-validation did not gradually increase modeling performance on the test set (see ESM Fig. 1) indicating that the root cause of quality issues leading to audit or inspection findings were rarely represented in the data. The biggest modeling improvement would be achieved if we were able to generate more process-related features. Potential sources for these features are listed in “Proposed Strategies to Improve the Model” section.

During feature engineering we limited ourselves to features that were associated with at least 5% (35 features in total) of all audits and inspections. If we had data from more audits and inspections we could potentially lower that threshold. We would also benefit from having larger test sets, which would allow for a finer graded calibration model.

We have reduced the modeling challenge to a binary classification problem ignoring that it is common to have more than one finding per CIF at a typical audit or inspection. For the business question at hand the binary outcome (absence or presence of a finding) was more important. A valid future approach could be to try modeling approaches that put more weights on audits and inspections with 2 or more findings compared to those with only one finding by oversampling or switching to a Poisson regression.

Due to the binary nature and the small number of our features and the limited number of observations in our test sets we did not manage to fit a smooth calibration curve. However, we were still able to define adequate upper and lower boundaries for our model outputs with a reasonable correlation between predicted and observed probabilities within those limits. As we add more features and more auditing data in the future the calibration curve will improve as well. Our data set was heavily biased by the Roche/Genentech product portfolio [18] with an overrepresentation of US-located sites conducting oncology studies. Our quality professionals were consistently reporting cultural differences in GCP implementations, which we can neither confirm nor deny due to the under-representation of non-western countries in our data set. Our analysis could definitely be improved if our training data set was more balanced.

Furthermore, building a consistent data set spanning 8 years merging data from many different internal data sources was quite challenging and the result was a compromise of keeping as many observations as possible and limiting the amount of missing feature values (see ESM Fig. 2). We will be able to assess additional features to the models as we continue our effort to gather a more complete data set as listed below, but which was not available at the time of this analysis.

### Proposed Strategies to Improve the Model

We used the data available as noted in ESM Table 1 as this data was readily available. In an ongoing effort to improve our modeling strategy, we will add new features to each of the CIF models with data not available at the time of this project, which could include the following:

- ICF complexity and length,
- Total number of subject re-consents,
- Delays in approvals from IRB/IEC of the consent forms,
- Number of translations for each consent form,
- System and study design complexity (number of eCRF pages and system interfaces with vendors),
- Complexity of dosing regimen,
- Site and study staff turnover.

### Calling for Cross-Company Collaboration and Data Sharing

Robust and data-driven quality assurance for clinical trials is a common interest across the pharmaceutical industry. However, the limited quality data sets of each individual company rarely provides enough signal for a consolidated risk analysis. Our analysis could greatly be improved if we had access to quality and operational data from other sponsors and would like to encourage sharing of non-competitive quality data in the GCP space.

We believe that collaboration and access to data from other sponsors could support further validation of our model. It would confirm or dismiss the hypothesis that clinical quality issues can be predicted, despite the inherent randomness and noise of clinical trials quality and operational data.

Although data sharing across sponsors remains fairly restricted [19], initiatives such as TransCelerate Placebo Standard of Care (PSoC) [20] have demonstrated value in a variety of use cases. In an upcoming collaboration with TransCelerate/Roche representatives and other pharmaceutical sponsors, we will propose a business use case to share historical clinical quality and operational study data. It would for example enable participating sponsors to further validate statistical models for clinical quality, contributing to enhanced patient safety and smart clinical trials execution.

## Conclusion

In this paper we laid the foundation for GCP quality risk modeling showing that even with a small data set at hand we could lay out the groundwork for a data-driven GCP risk assessment. Augmenting our internal data sets and cross-industry data-sharing initiatives can make this approach even more valuable. The model is now being piloted by Quality Program Leads at Roche/Genentech to complement our study/site risk assessment. This is part of a broader effort at Roche/Genentech Product Quality to leverage advanced analytics to augment and complement traditional clinical QA approaches. With regards to the model itself, there are plans to enhance it in the coming months (see “Proposed Strategies to Improve the Model” section). We will also submit a business use case through TransCelerate for sharing non-competitive operational and quality data while strengthening our collaboration with other pharmaceutical sponsors in this area.

## Electronic supplementary material

Below is the link to the electronic supplementary material.

Supplementary file1 (PDF 739 kb)
