# Supplementary material for: Harnessing the Power of Quality Assurance Data: Can We Use Statistical Modeling for Quality Risk Assessment of Clinical Trials?
Source: Ther Innov Regul Sci. 2020 Mar 30;54(5):1227–35. doi: 10.1007/s43441-020-00147-x (PMC7458946; doi:10.1007/s43441-020-00147-x)
Supplement: Supplementary file 1 — Supplementary file1 (PDF 739 kb) [file 43441_2020_147_MOESM1_ESM.pdf]

Harnessing the power of quality assurance data - can we use statistical modeling for quality risk assessment of clinical trials?

Therapeutic Innovation & Regulatory Science

Björn Koneswarakantha[1], PhD • Timothé Ménard[1], PharmD • Donato Rolo[2], MSc • Yves Barmaz[1], PhD • Rich Bowling[3]

1. 1F.Hoffmann-La Roche AG, Basel, Switzerland
2. Roche Product Ltd, Welwyn Garden City, United-Kingdom
3. Genentech Inc, South San Francisco, United States of America

Corresponding author: Timothé Ménard, timothe.menard@roche.com, F. Hoffmann-La Roche, CH-4070 Basel, Switzerland

In this ESM document we provided a detailed list of features and how they were used (Table 1). The coefficients and AUC values of the time series cross validation iterations (Figure 1) and an overview of the distribution of missing data in our data set (Figure 2).

Electronic Supplementary Material Table 1

Feature	Signal Detected in EDA	Used for modeling	Binary Feature	Frequency of Audits and Inspections
# Adverse Events per Visit at Site	yes	no	-	-
# Adverse Events with Reporting Delay (reporting date - start date) > 30 days at Site	no	-	-	-
# Adverse Events with Reporting Delay (reporting date - start date) > 90 days at Site	no	-	-	-
# Deaths per Visit at Site	no	-	-	-
# Major Protocol Deviations at Site	yes	no	-	-
# Minor Protocol Deviations at Site	yes	no	-	-
# Roche Trials Run in Same Therapeutic Area	no	-	-	-
# Screening Failures	no	-	-	-
# Serious Adverse Events per Visit at Site	yes	no	-	-
# Site Patients Enrollment	yes	yes	patient at site > 32	0.062
# Site Patients Enrollment	yes	yes	patient at site < 5	0.143
# Site Patients Screened	yes	no	-	-
# Special Interest Adverse Events at Site	no	-	-	-
# Target Countries Study	no	-	-	-
# Target Enrollment Study	no	-	-	-
# Target Sites Study	no	-	-	-
Blinding (double, open, single)	yes	no	-	-
Comparison (active, non-, placebo)	yes	no	-	-
Country of Site	yes	yes	USA site	0.24
Disease Type	yes	yes	cancer study	0.574
Disease Type	yes	yes	neurological or psychiatric study	0.1

Feature	Signal Detected in EDA	Used for modeling	Binary Feature	Frequency of Audits and Inspections
Disease Type	yes	yes	autoimmune study	0.111
Mean Adverse Event Reporting Delay (reporting date - start date) at Site	yes	yes	mean AE reporting delay < 31 days	0.072
Mean Adverse Event Reporting Delay (reporting date - start date) at Site	yes	yes	mean AE reporting delay > 227 days	0.043
Mean Adverse Event Reporting Delay (reporting date - start date) at Site	yes	yes	mean AE reporting delay 31 - 66 days	0.124
Molecule Class (kinase inhibitor, antibody, other)	yes	no	-	-
Pediatric	yes	yes	pediatric study	0.066
Randomization	yes	yes	non-randomized parallel or sequential study	0.078
Randomization	yes	yes	Non-randomized single group study	0.154
Ratio Screening Duration Actual/Commitment (months)	yes	no	-	-
Site Commitment to First Patient Screened Duration (months)	no	-	-	-
# Parallel Roche Studies in same Therapeutic Area	yes	yes	site burden score > 0.8	0.211
# Parallel Roche Studies in same Therapeutic Area	yes	yes	site burden score 0.25 - 0.47	0.192
# Parallel Roche Studies in same Therapeutic Area	yes	yes	site burden score < 0.25	0.159
Site Screen Failure Rate	yes	yes	screen failure rate site > 0.62	0.065
Site Screening Duration (months)	yes	yes	screening duration per patient > 3.1 months	0.065
Study Design (single group, parallel, sequential)	yes	yes	non-randomized parallel or sequential study	0.078
Study Design (single group, parallel, sequential)	yes	yes	Non-randomized single group study	0.154
Study Phase	yes	yes	phase I study	0.065

Electronic Supplementary Material – Figure 1

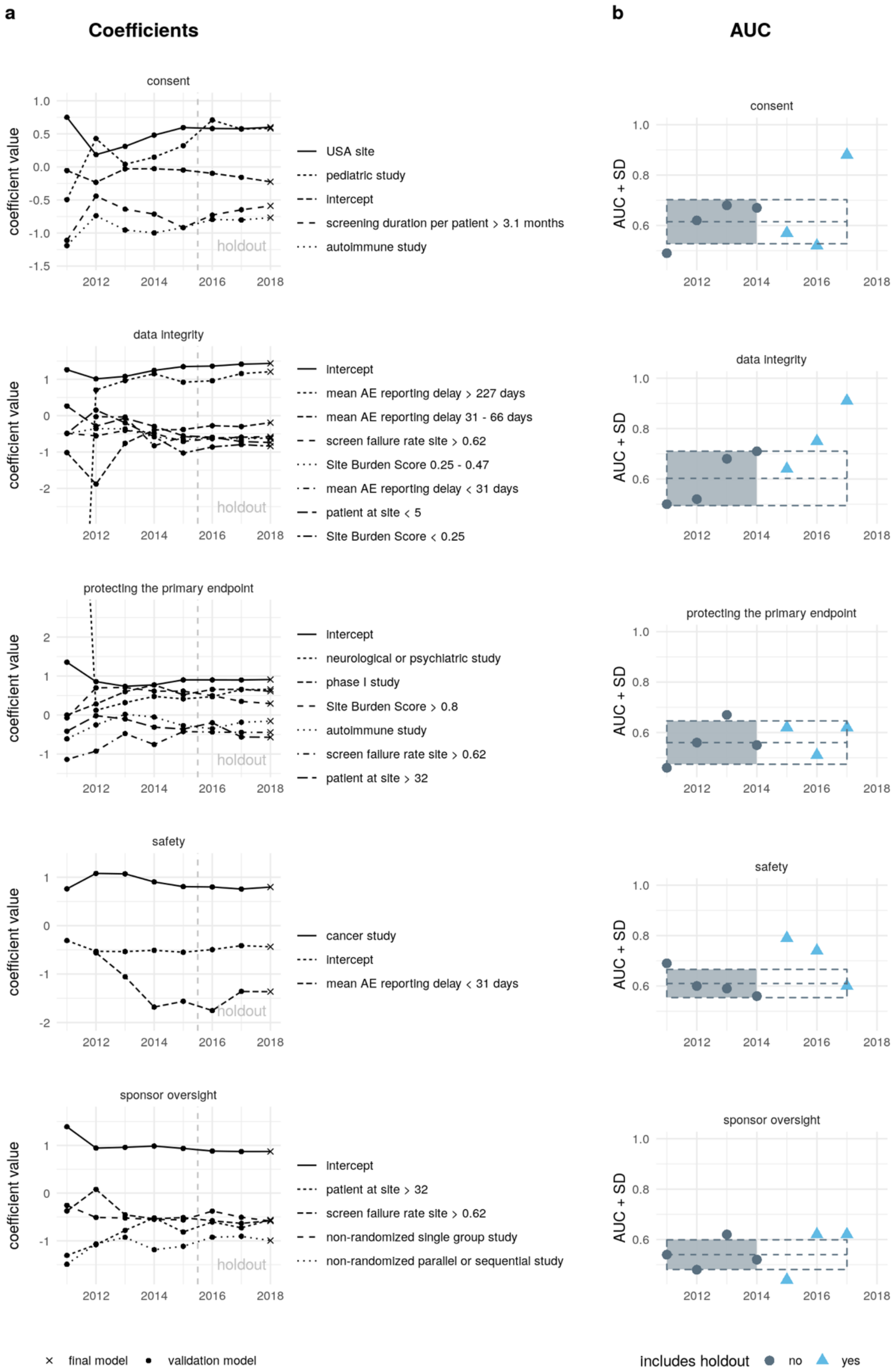

ESM Figure 1

Logistic regression coefficient values of all models fit during time series cross validation on the training sets including the final model. Trainings sets for models representing the 2014-2018 holdout time period (marked by the dashed line) including data that was not used for feature selection (a). Individual AUC values derived from predictions of the individual time series cross validation test sets have been plotted. The standard deviation range of AUC values derived from models that were not fitted on holdout data (2011-2014) is indicated as a dashed box. AUC values from 2015-2018 derive from models that included holdout data in their training set.

Electronic Supplementary Material – Figure 2

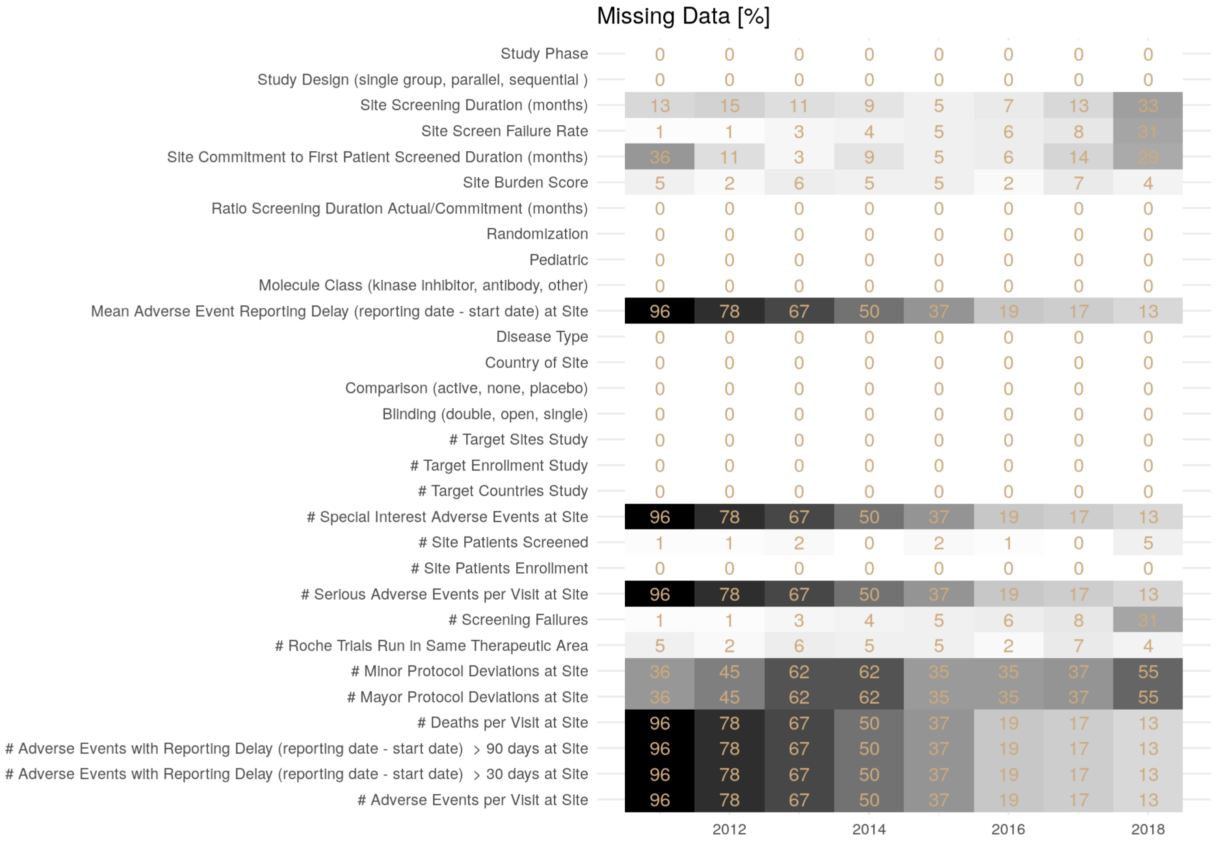

ESM Figure 2

Percentage of audits and inspections with missing values in a given feature in a given year. During feature binarization all missing values were replaced with ‘no’.
